# Potential Risk of Regional Disease Spread in West Africa through Cross-Border Cattle Trade

**DOI:** 10.1371/journal.pone.0075570

**Published:** 2013-10-09

**Authors:** Anna S. Dean, Guillaume Fournié, Abalo E. Kulo, G. Aboudou Boukaya, Esther Schelling, Bassirou Bonfoh

**Affiliations:** 1 Department of Epidemiology and Public Health, Swiss Tropical and Public Health Institute, Basel, Switzerland; 2 University of Basel, Basel, Switzerland; 3 Veterinary Clinical Sciences Department, Royal Veterinary College, University of London, United Kingdom; 4 Ecole Supérieur d’Agronomie, Université de Lomé, Lomé, Togo; 5 Direction de l’Elevage, Ministère de l’Agriculture, de l’Elevage et de la Pêche, Lomé, Togo; 6 Centre Suisse de Recherches Scientifiques en Côte d’Ivoire, Abidjan, Côte d’Ivoire; Auburn University, United States of America

## Abstract

**Background:**

Transboundary animal movements facilitate the spread of pathogens across large distances. Cross-border cattle trade is of economic and cultural importance in West Africa. This study explores the potential disease risk resulting from large-scale, cross-border cattle trade between Togo, Burkina Faso, Ghana, Benin, and Nigeria for the first time.

**Methods and Principal Findings:**

A questionnaire-based survey of livestock movements of 226 cattle traders was conducted in the 9 biggest cattle markets of northern Togo in February-March 2012. More than half of the traders (53.5%) operated in at least one other country. Animal flows were stochastically simulated based on reported movements and the risk of regional disease spread assessed. More than three quarters (79.2%, range: 78.1–80.0%) of cattle flowing into the market system originated from other countries. Through the cattle market system of northern Togo, non-neighbouring countries were connected via potential routes for disease spread. Even for diseases with low transmissibility and low prevalence in a given country, there was a high risk of disease introduction into other countries.

**Conclusions:**

By stochastically simulating data collected by interviewing cattle traders in northern Togo, this study identifies potential risks for regional disease spread in West Africa through cross-border cattle trade. The findings highlight that surveillance for emerging infectious diseases as well as control activities targeting endemic diseases in West Africa are likely to be ineffective if only conducted at a national level. A regional approach to disease surveillance, prevention and control is essential.

## Introduction

Animal movements within countries and across borders can facilitate the rapid spread of pathogens across large distances [1]. Recent examples include the spread of highly pathogenic avian influenza H5N1 globally [2] and of *Trypanosoma brucei rhodesiense*, one of the agents of Human African Trypanosomiasis, through cattle trade in Uganda [3]. In industrialised countries, this risk can be mitigated through strict importation controls. However, in developing countries, cross-border trade of live animals is often an important component of livestock production systems from both an economic and cultural perspective.

In West Africa, long-distance, cross-border cattle trade was estimated to be worth US$150 million in 2000 [4]. Traditionally, livestock have been raised in the semi-arid Sahel zone and traded with countries in the southerly forested zones [4], [5]. These cross-border movements continue to occur frequently and often outside of official veterinary control. Similar trends exist in East Africa, where cross-border trade involving Somalia, Ethiopia, Sudan, Kenya, and Tanzania was estimated to be worth US$61 million per annum in 2009, with only 10% of trade occurring through official channels [6]. In South-East Asia, interviews with cattle traders uncovered trade routes involving Thailand, Laos, Cambodia, and Vietnam. Almost half (45%) of the 60 Cambodian traders interviewed admitted to trading animals which they suspected to be infected with Foot-and-Mouth Disease (FMD) virus [7].

Understanding the potential pathways and risk for regional spread of infectious diseases is essential for tailoring appropriate control interventions in West Africa. In this context, a questionnaire-based survey was undertaken with cattle traders operating in cattle markets in northern Togo in 2012, and the potential risk of regional disease spread through trade routes was assessed through stochastic simulations.

## Methods

### Ethics Statement

This research was a component of a larger study of zoonotic disease epidemiology in Togo [8], and was approved by the Ethics Committee for Health Research (Comité de Bioéthique pour la Recherche en Santé) of the Ministry of Health of Togo. In Switzerland, approval was given by the Ethics Commission of the Cantons of Basel-Stadt and Basel-Land and the Research Commission of the Swiss Tropical and Public Health Institute of Basel, Switzerland. The information to be communicated to participants was provided as a written document to the interviewers and they received training regarding the consent process. Prior to interviewing, the study objectives, procedures and questionnaire content were explained to participants in their local language and they were assured that the questionnaire data would be treated anonymously. As the interviews were conducted in the cattle traders’ busy, outdoor workplaces, obtaining written consent was determined to be impractical in this setting. Similar to previous cross-sectional surveys conducted with traders in marketplaces [9], [10], all participants provided informed verbal consent before the interview, as approved by the aforementioned ethics and research commissions. The informed consent of each participant was recorded by the interviewer on the questionnaire form at the time of interview, and refusals to participate were recorded on a separate sheet.

### Study Site

The study was conducted in the northernmost region of Togo, the Savannah Region, which is bordered by Burkina Faso, Ghana, and Benin. The Savannah Region is a pastoral zone important for livestock raising, with approximately half of Togo’s cattle population found in this region, estimated to be 138,000 in 2011 (Direction de l’Elevage - Togo, personal communication). The area also receives a large number of transhumant (i.e. semi-nomadic) herds each dry season, the official period of transhumance being from January-May. These herds are mainly from Burkina Faso, as well as from Benin and Niger.

### Questionnaire Survey

Through discussion with regional veterinary services and livestock traders, the nine biggest cattle markets in the region were identified. Markets were open 1–2 days per week. The target population was traders of live cattle operating in markets in northern Togo. The survey was conducted in February-March 2012 and, in order to capture as many traders as possible, larger markets were visited up to 5 times.

Structured questionnaire-based interviews were conducted by two trained interviewers. Although the questionnaire was in French, the official language of Togo, interviews were also conducted in four local languages. Traders were asked to name all of the sites that they visited to purchase or sell cattle during the current dry season and the previous wet season. The information recorded by the interviewers included the nature of the site (market or informal trading place), its full location (village, district, province and country), and the type of the stakeholders with whom they were trading (such as traders, farmers, or butchers). If the traders reported visiting markets, the frequency of their visits was recorded. For each location, traders were asked to specify the minimum and maximum number of cattle sold or purchased per month, if they visited that location every month in a given season. If a location was visited only sporadically, they instead specified a minimum and maximum per season. Additionally, traders were asked whether they sold animals from, or bought animals for, herds that they personally owned. The locations of these herds, the minimum and maximum number of cattle sold/purchased and the frequencies of sales/purchases were recorded. As the definition of dry and wet seasons may vary between individuals, participants were asked to define the months corresponding to these periods.

Additionally, the manager of each market was asked to estimate the number of traders operating in the market each open day in both seasons. In order to minimise data entry errors, all data were entered twice into a pre-designed Microsoft Access 2003 database and cross-checked for discrepancies using EpiInfo 3.5.3 (Centers for Disease Control and Prevention, USA).

### Market Catchment Area

Locations where cattle were bought or sold by traders operating in the Savannah market system were visualised using MapInfo Professional Version 7.0. The centroid of each province was plotted in order to show the geographic distribution.

### Cattle Flows Through the Market System

The analysis was conducted using R statistical software Version 2.12.2 [11]. Characterisation of the flows of cattle into and out of the market system of the Savannah Region could not be directly deduced from the empirical data, due to two constraints. Firstly, it was not possible to sample every trader operating in the market system during the course of the survey. Secondly, although the number of cattle purchased and sold in each location was known for each trader, information about the actual origin or destination of these animals was missing. In other words, the number of animals purchased by a trader from locations A and B was known, as was the number sold to locations C and D, but the proportions of cattle purchased in A or B that were then sold to C or D were unknown. Consequently, it was necessary to estimate the flows of cattle into and out of the Savannah market system through stochastic simulations. The simulation algorithm is described below, which was repeated over 10,000 simulations.

In order to assess the proportion of the total trader population in the Savannah market system that was captured by the survey, the number of trader-days per season per market was first calculated from the market visit frequencies reported by the traders. This was compared with the estimate of the number of traders provided by the market manager.

If the trader interview data gave a lower estimation of the number of trader-days per season than the market manager’s estimation for a given market, it was assumed that the trader sample did not capture the full trader population of the market. In these markets, the trader data were completed by randomly re-sampling from the group of traders visiting this market until the number of trader-days estimated by the manager was reached. This modified dataset was then used to reconstruct the flow of cattle between locations.

Cattle movements were simulated for each individual trader as follows. The minimum and maximum numbers of cattle reported to be sold or purchased at each site by a given trader were summed over one season (dry or wet). These minimum and maximum values accounted for the monthly variability reported by traders as well as the uncertainty associated with the recall of the number of cattle traded. As traders were often livestock owners who bought and sold cattle for their own herds, the location of their herd was considered as the sale location for cattle which they purchased for themselves. For those traders selling cattle from their own herds, the location of the herd was considered as the purchase location. For each trader *t*, the number 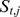 of cattle sold in location *j* (with 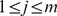) and the number 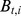 of cattle purchased in location *i* (with 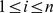) were then randomly drawn from their respective ranges extending from the minimum to maximum value. In order to ensure that a given simulated trader *t* sold as many cattle as he purchased over an entire season, the total number 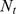 of cattle traded was randomly drawn from the range extending from the total of number sold, 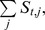 and the total number purchased, 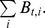 The simulated number of cattle sold and purchased in each location was then defined as 
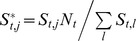
 and 
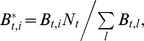
 respectively.

The sold animals were attributed to an origin by exploring two different scenarios, Location Scenarios 1 and 2. In the first scenario, Location Scenario 1, the probability of an animal being sold in any of the sale locations did not depend on its purchase location. For each animal sold by a trader, an origin was allocated by randomly sampling from the locations given for the animals purchased by this trader, without replacement. In other words, each animal sold by a given trader was randomly matched with a unique animal purchased by this trader. The flows of cattle resulting from each individual trader were then summed. However, given the possibility that cattle may have been more likely to be sold at a site closer to the purchase location, an assumption of dependence of purchase and sale sites was also explored. In this second cattle flow scenario, Location Scenario 2, cattle were preferentially sold in the same country as where they were purchased; or, for purchases in Togo, they were preferentially sold in the same administrative region.

For each simulation, the number of cattle entering the market system did not necessarily equal the number leaving. The number of cattle flowing through the Savannah market system in each simulation was, therefore, assumed to be the greater of these two values. After running the algorithm over 10,000 simulations, the mean, minimum and maximum numbers of cattle flowing between locations was assessed.

### Market Network

A network of contacts between markets in the Savannah Region was simulated using R package sna [12], with markets as nodes and animal movements as edges, which were simulated according to the algorithm described above. The network was directed, meaning that the direction of animal movements was accounted for. Each directed edge connecting two markets was given a weight, equal to the number of cattle traded between these two locations. Network connectivity was assessed via the giant strongly connected component (GSCC) and giant weakly connected component (GWCC). The GSCC refers to the part of the network within which all nodes can reach one another through directed paths, whereas the GWCC includes nodes that can reach one another through undirected paths. The in- and out-degree distributions of the binary and weighted networks were assessed. For the binary network, the in- and out-degree referred to the number of Savannah markets sending cattle to and receiving cattle from a given Savannah market, respectively. For the weighted network, the in-degree referred to the number of cattle being moved to a given Savannah market from other Savannah markets, with the out-degree being the converse.

Cattle in the network were moved directly from their place of purchase to their place of sale based on the results of the cattle flow simulations described above. It is possible, however, that a trader visiting several markets may conduct his visits in a particular order. For example, a trader may purchase all of his cattle in market A, move these cattle to market B where some would be sold, and then finally visit market C to sell those remaining. Information about the order of market visits was not available, but could influence the distribution of links between markets. In order to explore this, an algorithm which stochastically ordered the market visits of each trader was run over 1,000 simulations. Further information is given in a supplementary file, Text S1.

### Risk of Disease Spread Through Market System

The risk of cross-border disease spread through the simulated cross-border livestock flows was assessed for Location Scenario 1. The risk was defined as the probability of a disease invading an area *j* through importation of cattle from a disease-endemic area *i* within a one year period. The pathway was divided into two steps: firstly, the introduction of an infectious animal into area *j* from area *i*; secondly, the spread of disease within the cattle population of area *j* due to the introduction of this infectious animal. Given that border crossings rarely involve veterinary assessment, it is assumed that infectious animals would be able to cross into neighbouring countries without detection and quarantine. Therefore, the probability *p_i_* of exporting an infectious animal from an area *i* can be approximated by the prevalence of the disease in area *i*. The number 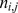 of animals moved from area *i* to *j* within the one year period was taken from the results of the simulations previously described.

When an infectious animal has been introduced into an area *j*, the disease may either fade out or spread within the cattle population of area *j*. The basic reproduction number of a disease (*R_0_*) is the number of secondary cases resulting from the introduction of one infectious case into a fully susceptible population. It refers to a pathogen’s potential to spread in a given population. The cattle population in area *j* was assumed to be fully susceptible and to mix homogeneously. With 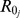 being the reproduction number in area *j*, the likelihood of disease extinction soon after the introduction of one infected animal into this population was equal to 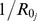 [13], and the probability of a sustained outbreak was 
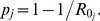
 The risk *P* of disease invasion was therefore:
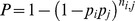



By varying 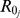 and 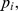 the risk of an exotic disease invading the cattle population of the Savannah Region in Togo through cattle trade from Burkina Faso was explored. Here, 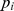 refers to the disease prevalence in Burkina Faso, and 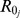 refers to the potential of a disease to invade the Savannah cattle population following the introduction of an infectious animal into one of its herds through the Savannah market system. The Savannah cattle population was assumed to mix homogeneously.

Additionally, the probability of a disease invading at least three other countries through cattle trade from Savannah herds was investigated. Here, 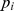 refers to the disease prevalence in the cattle population in the Savannah region, and 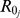 refers to the potential of a disease to invade a cattle population in a given country after the introduction of an infectious animal from the Savannah Region into the market system of this country. 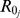 was assumed to be the same in all countries trading with the Savannah Region and, in each given country, the local cattle population and cattle traded through the local market system were assumed to mix homogeneously. This assumption was necessary because data relating to flow of cattle from the market systems into herds in these countries were not available.

## Results

### Descriptive Analysis of Empirical Data

Two hundred and twenty-six traders were interviewed, with a refusal rate of 12%, mainly due to lack of time to participate. In each of the nine markets, 9–55 traders were interviewed (median 20, IQR: 11–36). For three markets, the number of trader-days calculated from the trader interview data accounted for only 24–77% of the trader-days estimated by the market manager, requiring modification (inflation) of their datasets. For the other six markets, the market manager estimated less trader-days than the trader interview data and modification of the dataset was not required. Only 8.4% of traders (19 of 226) transported their animals between locations solely by vehicle, with the remainder herding their animals by foot, either exclusively or in combination with road transport. The majority of traders defined the dry season as extending from October – April, and the wet season from May – September.

Given the higher number of cattle traded at a greater number of locations during the dry season compared to the wet season, these data will be presented in detail below. The corresponding information for the wet season is provided in a supplementary file, Data S1. Most interviewees (193/226) not only acted as traders, but also bought and sold cattle for their own private herds. However, the proportions of their purchases and sales that involved their own herds were small. In the dry season, the median proportion of their purchases that represented cattle taken from their own herds was only 1.1% (IQR: 0.7–1.8%), and the proportion of their sales corresponding to cattle being added to their own herds was 1.5% (IQR: 1.0–2.3%). In the dry season, cattle were sold from 179 herds. Most of these herds (146 of 179) were located in the Savannah Region, as well as other areas of Togo (5 of 179), Burkina Faso (23 of 179) and Ghana (5 of 179). Similarly, most of the 175 herds which received purchased cattle during the dry season were in the Savannah Region (141 of 175), as well as other areas of Togo (5 of 175), Burkina Faso (23 of 175), Ghana (4 of 175), Benin (1 of 175) and Niger (1 of 175).

The distribution of the average number of cattle traded per trader during the dry season was right-skewed. While the median was 500 (IQR: 173–639), as many as 2,916 cattle were reported to be traded by a single trader during the season. The median numbers of purchase and sale locations of the traders were 4 (IQR:3–5) and 3 (IQR:2–4), respectively, regardless of season, with a maximum of 8. Most of these sites were cattle markets within the Savannah Region. The median number of Savannah markets in which traders operated for purchase or sale was 3 (IQR: 2–4) in the dry season.

Nearly three quarters of traders (166 of 226, 73.5%) in the dry season bought cattle in at least one Savannah market and sold cattle in at least one other Savannah market. Around half of the cattle purchases and sales reported by traders (55.2% and 51.4%, respectively) took place in Savannah markets, whilst 35.8% and 21.6% of purchases and sales, respectively, took place in another country. Of all the cattle purchases and sales taking place in the 28 Savannah markets, 83% and 82% respectively took place in only 4 markets. Outside of Togo, most of the foreign cattle purchases (85.3%) were conducted in Burkina Faso, and 38.4% of foreign cattle sales took place in Nigeria. The number of traders operating at different purchase and sale locations as well as the numbers of cattle traded at these sites are summarised for the dry season in Table 1.

**Table 1:** Empirical data from interviews with 226 cattle traders – dry season.

	Purchase and sale locations									
	Savannah markets	Savannah herds	Savannah butchers	Other Togo markets	Other Togo herds	Benin	Burkina Faso	Ghana	Niger	Nigeria
No. of traders purchasing or selling*	226 (100%)	172 (76.1%)	39 (17.3%)	91 (40.3%)	7 (3.1%)	37 (16.4%)	81 (35.8%)	22 (9.7%)	2 (0.09%)	31 (13.7%)
No. of traders purchasing*	202 (89.4%)	163 (72.1%)	0	16 (7.1%)	6 (2.7%)	10 (4.4%)	79 (35.0%)	11 (4.9%)	0	0
No. of traders selling*	190 (84.1%)	152 (67.3%)	39 (17.3%)	80 (35.4%)	5 (2.2%)	34 (15.5%)	32 (14.2%)	18 (8.0%)	2 (0.09%)	31 (13.7%)
No. of cattle purchased, ranging from min. to max.°	58334–66285 (55.2%)	6605–7370 (6.2%)	0	2761–3094 (2.6%)	299–327 (0.3%)	2480–2908 (2.4%)	32776–36067 (30.5%)	3120–3391 (2.9%)	0	0
Median proportion (%) of total cattle purchased per trader+ (IQR)	77.0 (53.0–99.0)	2 (1.0–18.5)	0	28.5 (19.0–34.5)	2.5 (1.3–13.5)	33.5 (27.0–38.0)	55 (29.0–95.0)	29 (18.5–49.5)	0	0
No. of cattle sold, ranging from min. to max.°	54675–61312 (51.4%)	2519–2806 (2.4%)	2961–3388 (2.8%)	23177–26084 (21.8%)	67–72 (0.1%)	6422–6997 (5.9%)	2933–3533 (2.9%)	4796–5282 (4.5%)	36–36 (<0.1%)	8779–9914 (8.3%)
Median proportion (%) of total cattle sold per trader+ (IQR)	83.5 (50.0–99.0)	2.0 (1.0–4.0)	15.0 (10.0–22.0)	45.0 (30.8–61.3)	2.0 (1.0–2.0)	26.5 (21.0–32.8)	5.0 (1.0–27.0)	26.0 (20.3–39.3)	6.5 (4.3–8.8)	28.0 (19.0–35.0)

* The numbers of traders purchasing from, and selling to, different locations over the dry season are shown. The proportion of the total number of traders interviewed is given in brackets as a percentage. Markets and herds located outside of the study zone, the Savannah Region, are referred to as “Other Togo markets” and “Other Togo herds”, respectively. No butchers were located outside of the Savannah Region.

°The minimum and maximum numbers of cattle purchased and sold in these locations are presented for the dry season, expressed in brackets as percentages of the average number of cattle purchased or sold.

+ This is the median value of the proportion of each trader’s purchases or sales taking place in the given locations, expressed as a percentage. The interquartile range (IQR) is given in brackets. Traders that did not purchase in a given location were excluded.

**Figure 1:**
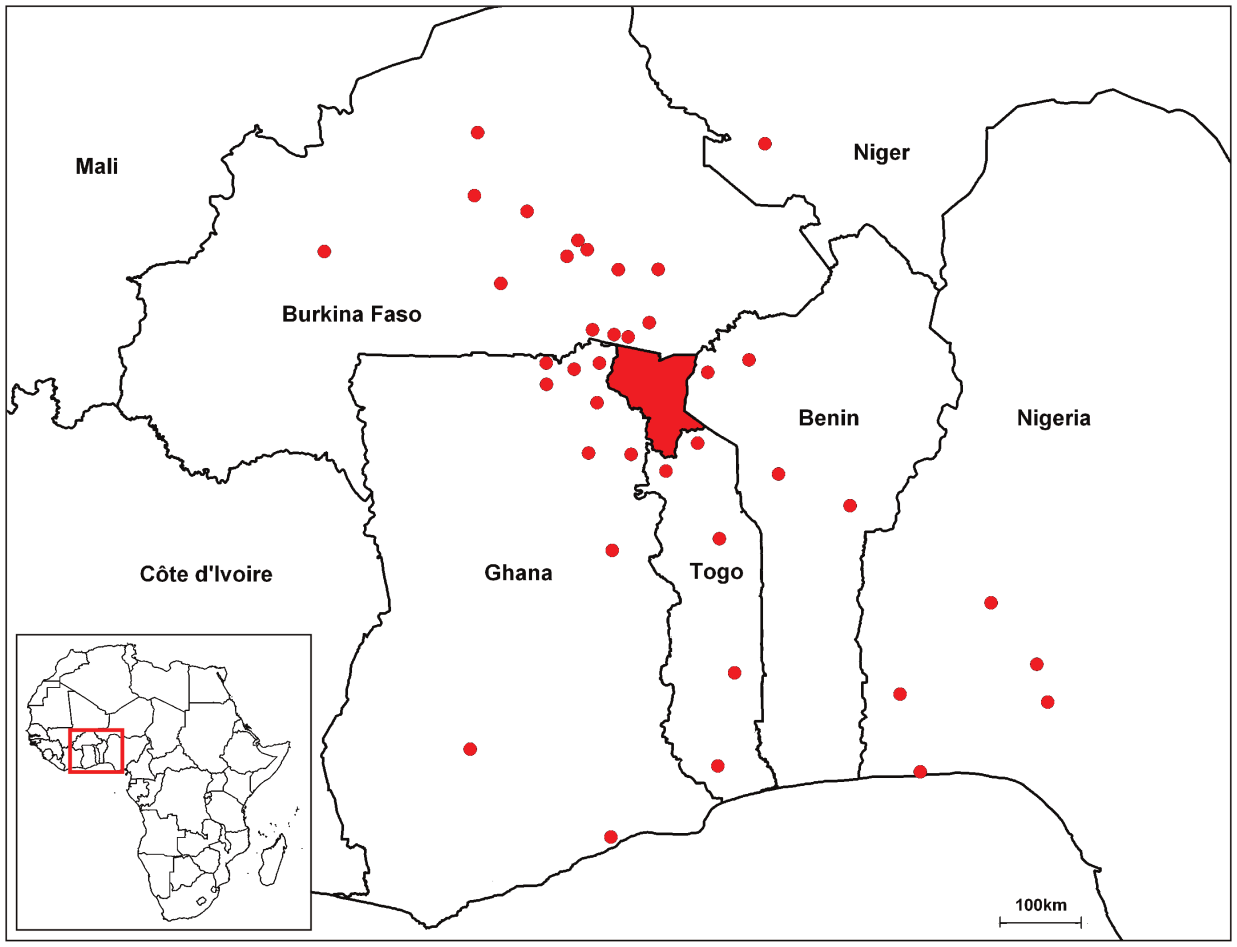
Study zone in West Africa. The study zone, the Savannah Region of northern Togo, is shaded red. The centroids of the other districts where the interviewed traders also bought or sold cattle are shown as red dots.

The market catchment area is shown in Figure 1, with traders operating in the Savannah market system also operating in Burkina Faso, Ghana, Benin, Nigeria, and Niger, as far as 500 km from the Savannah Region. Overall, more than half of the traders (121 of 226, 53.5%) operated in at least one other country outside of Togo. Among those traders operating in multiple countries, only one quarter (32 of 121, 26.4%) conducted both purchase and sale activities in at least two countries. In the dry season, almost two thirds of the traders operating in Burkina Faso (49 of 81, 60.5%) conducted only purchases in Burkina Faso, without any sales. Half (11 of 22, 50.0%) of traders operating in the dry season in Ghana, three quarters (27 of 37, 73.0%) of traders operating in Benin and all (31 of 31, 100%) of the traders operating in Nigeria only sold cattle in these countries, without purchasing.

### Simulated Livestock Flows

The 10,000 model simulations gave means of 32,370 cattle (range: 31,070–34,180) flowing into the Savannah market system during the dry season and 28,860 (range: 27,940–29,970) during the wet season. Location Scenario 2 with non-independence of buying and selling locations did not have a notable impact on the results, with a mean cattle flow into the Savannah market system during the dry season of 28,260 (range: 26,810–29,500) and 24,900 (range: 23,670–25,930) in the wet season. Overall trends of animal flows were the same regardless of season. Given the greater cattle flows during the dry season, these data will be presented in detail below. Results from the wet season are provided in a supplementary file, Data S1. A summary of results for Location Scenarios 1 and 2 for both the dry and wet seasons is provided as a table in a supplementary file, Data S2.

**Figure 2:**
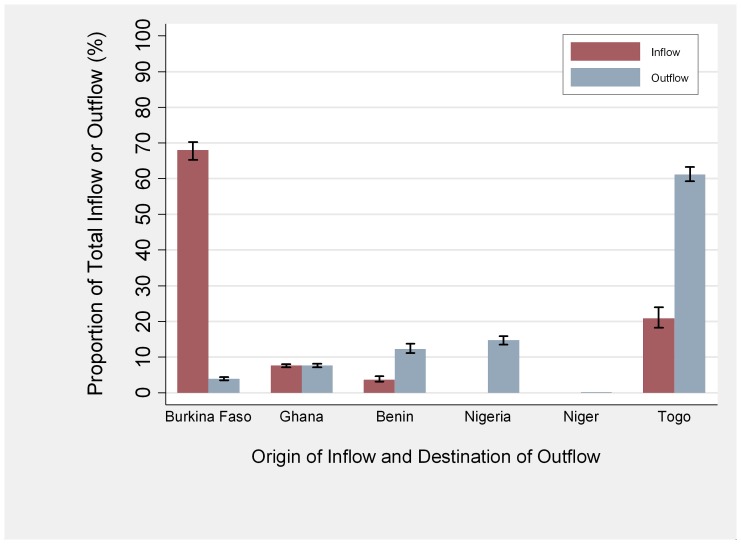
Simulated flows of cattle into and out of the market system of Savannah Region, Togo. These simulated mean flows are shown for the six month dry season period, as a proportion of total flow into or out of the Savannah market system. The range of simulated values from minimum to maximum are shown as black bars.

Figure 2 shows the mean proportions of animals entering into and leaving the Savannah market system obtained by the simulations for the Location Scenario 1 for the dry season. More than three quarters (79.2%, range: 78.1–80.0%) of cattle flowing into the Savannah market system during the dry season originated from other countries, with 68.0% (range: 65.2–70.2%) of inflow coming from Burkina Faso, 7.6% (range: 7.2–8.0%) from Ghana and 3.6% (range: 3.1–4.7%) from Benin. Half of the cattle leaving the Savannah market system in the dry season (49.3%, range: 47.0–51.7%) were sent to Togolese markets outside of the Savannah Region. The majority (93.7%, range: 92.7%–94.5%) of these were sent to one large market near the coastal capital city of Lomé, where animals are generally slaughtered for meat consumption. One third of the cattle (38.8%, range: 36.7–40.7%) leaving the Savannah market system moved into other countries, with 7.8% (range: 7.3–8.4%) of outflow into Ghana, 12.3% (range: 11.1–13.8%) into Benin and 14.7% (range: 13.5–15.9%) into Nigeria. The results of Location Scenario 2 did not demonstrate any major differences in flow, as detailed in Data S2.

In the dry season, 2,992 (range: 2775–3284) cattle flowed into herds in the Savannah Region, equating to 2.2% (range: 2.0–2.4%) of the estimated total cattle population size in the Savannah Region. Given that most herds likely breed their own replacement animals, many more animals flowed in the reverse direction from Savannah herds into the market system. There was a mean of 13,633 cattle (range: 9,096–24,506) leaving herds in the dry season, equating to 9.9% (range: 6.6–17.8%) of the estimated total cattle population size. Location Scenario 2 produced similar results with 2,989 cattle (range: 2,733–3,305) flowing into herds and 13,730 (range: 9,408–23,940) leaving herds. Flows into and out of the Savannah herds followed the same trends as the aforementioned market system flows.

### Market Network during Dry Season

In the dry season, the market system consisted of 28 markets. They formed a well connected network incorporating all but one of the markets. The GWCC, estimating the upper bound of the maximum epidemic size, was 27. Nearly half of these markets (13) formed the GSCC, estimating the lower bound of the maximum epidemic size. When using the alternative algorithm for reconstructing the order of market visits, the GSCC was even higher, with a median of 20 markets (range: 14–23). Further details of this algorithm are provided in a supplementary file, Text S1.

**Figure 3:**
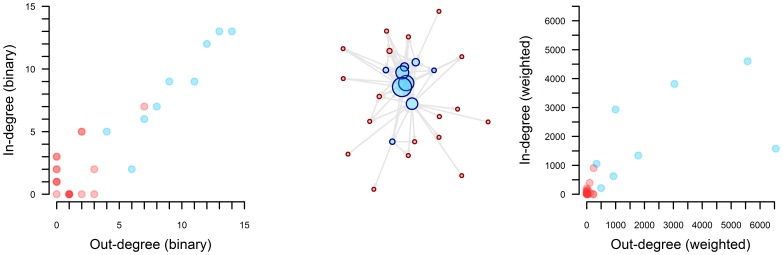
Market network of the Savannah Region of Togo and degree distributions. The graph on the left shows the binary in-degree as a function of the binary out-degree, and the graph on the right shows the weighted in-degree as a function of the weighted out-degree, during the dry season. The 9 markets where the survey was conducted are coloured blue and the other 19 are red.

Figure 3 shows the distribution of markets during the dry season as a function of their binary and weighted in- and out-degrees. The majority of markets (17 of 28) received cattle from at least two other markets, with a maximum of 13 other markets. Approximately half of the markets (15 of 28) sent cattle to at least two other markets, with a maximum of 14 other markets. However, most cattle movements within the Savannah market network were mediated by a small number of markets: 4 markets accounted for 73.7% and 78.6% of the total weighted in- and out-degrees, respectively. The markets with the highest degrees were those included in the survey, shown as blue circles in Figure 3. The Savannah market network for the wet season is presented in a supplementary file, Data S1.

### Disease Risk

**Figure 4:**
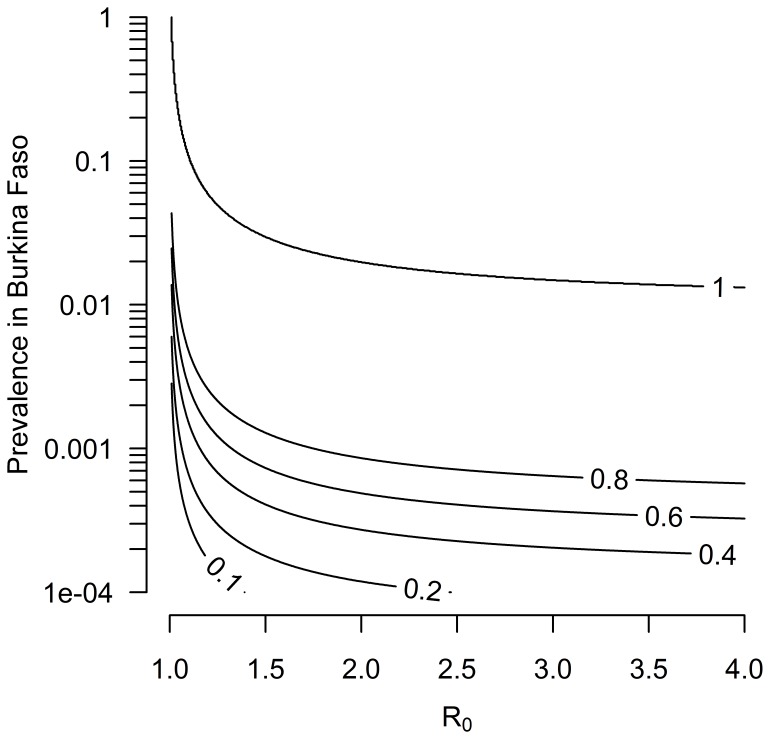
Probability of a disease invading the cattle population of the Savannah Region in Togo through cattle trade from Burkina Faso. The probability of a disease invading the cattle population of the Savannah Region is shown as a function of the disease prevalence in Burkina Faso and the basic reproduction number of the disease, R_0_. Here, R_0_ relates to the potential of a disease to invade the Savannah cattle population following the introduction of an infectious animal into one of its herds.

**Figure 5:**
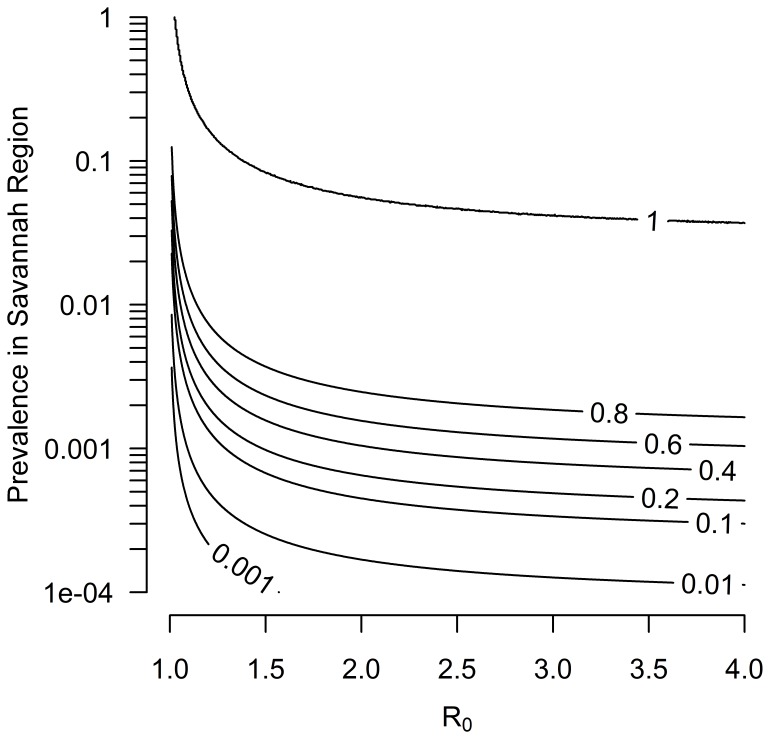
Probability of a disease invading at least three other countries through cattle trade from Savannah herds. The probability of disease invasion is shown as a function of the disease prevalence in the cattle population in the Savannah region and the basic reproduction number of the disease, R_0_. Here, R_0_ relates to the potential of a disease to invade a cattle population in a given country, after the introduction of an infectious animal into the market system of this country.

Figure 4 shows the average probability over the course of one year that a disease present in Burkina Faso will be introduced through the Savannah market system into Togolese herds and result in an outbreak. For a hypothetical disease, even at a low prevalence of less than 1% in Burkina Faso and low transmissibility with an R_0_ of around 1.25, there was a high probability (80%) of an outbreak in Togo. When disease prevalence is higher, between 1–10%, this probability reaches 100%. Similarly, if a hypothetical disease with an R_0_ of around 1.25 is present in Savannah herds at a prevalence of less than 1%, the probability of disease being introduced into at least 3 other countries in the region through the Savannah market system is also 80% (Figure 5).

## Discussion

Although livestock market networks and implications for disease spread have been described in detail in developed countries [14], [15], few data are available from developing countries and are not captured by official datasets such as FAOSTAT from the Food and Agricultural Organization of the United Nations (FAO) [16]. This study quantitatively captures the potential disease risk resulting from large-scale, cross-border cattle trade between Togo, Burkina Faso, Ghana, Benin, and Nigeria for the first time. The findings will serve as the basis for further research hypotheses and should lead to strengthened collaboration at the regional level in the planning of disease control measures. The number of animals flowing into the northern Togolese Savannah market system during the dry season equals nearly one quarter of the resident cattle population. Although more than half of these animals were reported by sellers as having been purchased in Burkina Faso, it is possible that some animals originated from further afield, such as Mali or Niger. Through the cattle market system of northern Togo, non-neighbouring countries are potentially epidemiologically connected via trade routes.

The GSCC of a network is an estimate of the lower bound of the maximum epidemic size for a given disease, whilst the GWCC estimates the upper bound [17]. The Savannah market network displayed high connectivity, with nearly half of the markets being incorporated into the GSCC and all but one forming the GWCC. In the alternative algorithm for reconstructing the sequence of market visits (Text S1), the GSCC was higher, incorporating the majority of markets. This suggests that a disease introduced into one market could rapidly spread to other markets. The market network of northern Togo is, therefore, a potential conduit for disease spread between West African countries. Although no data are available, it is likely that the scale of cross-border trade through the Togolese market network is not a unique scenario, but is rather the norm for West Africa.

These findings are relevant not only to the surveillance and control of newly emerging diseases, but also endemic diseases. Cross-border cattle trade and transhumance may have contributed to the genetic diversity of *Brucella abortus* strains circulating in the study zone [8]. These cross-border movements could also potentially explain the overlapping distribution of FMD serotypes O, A, SAT1 and SAT2 across West African countries [18]–[20], particularly given that wildlife play a less important role in disease transmission than in East Africa. The results of this study are of direct relevance to the effective implementation of the Global FMD Control Strategy of the FAO and the World Animal Organisation for Animal Health (OIE) announced in 2012, a 15 year program which seeks to reduce the global impact of this devastating livestock disease [21]. Estimates of the R_0_ of FMD in sub-Saharan Africa are not available. However, the R_0_ of Rinderpest, a viral disease of cattle officially eradicated in 2011, has been estimated to range between 1.2–4.4 in Somalian and Sudanese cattle populations [22], [23]. The R_0_ of Contagious Bovine Pleuropneumonia, a severe respiratory disease of cattle in Africa, is estimated to fall between 3.2–4.6 in pastoral herds of southern Sudan [24]. Therefore, the R_0_ range of 1–4 used in this study to assess the risk of disease spread through regional cattle trade in West Africa is appropriate.

In addition to animal movements through trade, the Savannah Region of northern Togo also receives a large number of transhumant herds from the Sahel zone in search of grazing pasture and water sources during the dry season. Members of the Economic Community of West African States (ECOWAS) are legally bound to permit seasonal cross-border movements of herds [25]. In 2011, 47,000 transhumant cattle entered the Savannah Region through official Togolese government check points (Direction de l’Elevage - Togo, personal communication), although it is likely that an even greater number entered the country unofficially. The risk of regional disease spread in West Africa through trade is, therefore, further compounded by transhumance. In northern Togo, there is evidence that anthrax outbreaks occur along the routes followed by transhumant herds. As many herds do not follow the official routes designated by the Togolese authorities, conducting disease surveillance in this zone is particularly challenging [26]. According to the market managers, the higher cattle flow through markets during the dry season reflected contributions from transhumant herds temporarily visiting Togo.

This study highlights the importance of a regional approach to disease control activities in West Africa. Prior to the annual childhood polio vaccination campaign implemented in 20 countries in Central and West Africa in March 2012, health care providers of border districts in the Savannah Region met and discussed with their counterparts in neighbouring countries. The animal health sector should also invest in cross-border disease prevention activities, such as synchronised vaccination campaigns or formal systems for the communication of unusual animal health events.

### Limitations

The questionnaire was written in French, the official language of Togo, Burkina Faso, and Benin. However, interviews were predominantly conducted orally in local languages by two trained multilingual interviewers. Occasionally, interpreters were also required. Due to the linguistic complexity of the study zone, data errors due to incorrect interpretation are possible. As the survey results are based on estimations of cattle transactions by traders, rather than witnessed transactions, there is a risk of recall bias.

The flow of cattle through the Savannah market system was reconstructed using simulations. While it is possible that the complexities of the market system have not been fully captured, these are unlikely to have a major impact on the results: estimates obtained when assuming independence or dependence of purchase and sale locations (Location Scenarios 1 and 2) were similar. This is likely due to the fact that when operating in locations outside of the Savannah region, most traders either purchased or sold cattle, not both. However, the data only capture cattle trade through the formal market system. Given that informal trade also occurs, the scale of cross-border cattle trade is likely to have been underestimated. Furthermore, small ruminant cross-border trade has not been considered in this study. Although further data collection would improve the accuracy and applicability of the study findings, substantial investment of resources would be required.

The order in which traders visited markets in the Savannah Region was not known and the network of cattle movements between these markets had to be reconstructed. While the GSCC size varied, it was always greater than 45% of all markets, meaning that all simulated networks displayed high connectivity. Moreover, most of the purchases and sales were always mediated by only a few markets. These dominant markets were those where the interviews were conducted. This may suggest that the sampling approach may have potentially introduced bias. However, given that these markets were identified by the local veterinary services as the largest markets in the Savannah Region, the constructed network may indeed reflect the true structure.

The estimations of the risk of disease spread through the market system assumed a homogenously mixing population, simplifying animal contact patterns and the underlying disease dynamics. Collecting further information on cattle population dynamics in this region would be useful for refining this estimate. Moreover, pathogen amplification within herds and within markets was not accounted for. If such amplification did occur, this would only serve to further increase the risk of disease spread.

### Conclusions

By stochastically simulating data collected by interviewing cattle traders in northern Togo, this study identifies potential risks for regional disease spread in West Africa through cross-border cattle trade. The findings highlight that surveillance for emerging infectious diseases as well as control activities targeting endemic diseases in West Africa, such as FMD, are likely to be ineffective if only conducted at a national level. A regional approach to animal disease surveillance, prevention and control is essential.

## Supporting Information

Data S1 **Detailed results for wet season.** Empirical data as well as results of the livestock flow simulations and network analysis are presented for the wet season, complementing the information provided in the manuscript for the dry season.
(DOC)

Data S2 **Summarised simulation results for wet and dry seasons.** The results of the 10,000 model simulations of livestock flows through the Savannah market system in the dry and wet seasons are provided in a table. This includes results for the independent and non-independent purchase and sale location scenarios, Location Scenario 1 and Location Scenario 2, respectively.
(DOC)

Text S1 **Accounting for the order of market visits in the analysis of the Savannah market network.** The process of stochastically ordering the market visits undertaken by traders in the Savannah market network is described for both dry and wet seasons.
(DOC)
