# Supplementary material for: Potential Risk of Regional Disease Spread in West Africa through Cross-Border Cattle Trade
Source: PLoS One. 2013 Oct 9;8(10):e75570. doi: 10.1371/journal.pone.0075570 (PMC3794041; doi:10.1371/journal.pone.0075570)
Supplement: Data S1 — Detailed results for wet season. Empirical data as well as results of the livestock flow simulations and network analysis are presented for the wet season, complementing the information provided in the manuscript for the dry season. (DOC) [file pone.0075570.s001.doc]

**Data S1: Detailed results for wet season**

**Wet season empirical data**

Of the 226 traders interviewed, 213 also traded in the wet season. Most interviewees (180 of 213) not only acted as traders, but also bought and sold cattle for their own private herds. However, the proportions of purchases and sales that involved their own herds were small. The proportion of purchases that represented cattle taken from their own herds was only 1.5% (IQR: 1.0-2.3%), and the proportion of sales corresponding to cattle being added to their own herds was 1.8% (IQR: 1.1-2.6%).

The distribution of the average number of cattle traded per trader was right-skewed. Compared to the dry season, less cattle were traded with a median of 333 (IQR: 167-660) and a maximum of 2,882. The median numbers of purchase and sale locations of the traders were 4 (IQR:3-5) and 3 (IQR:2-4), respectively, regardless of season, with a maximum of 8. Most of these sites were cattle markets within the Savannah Region. The median number of Savannah markets in which traders operated for purchase or sale was 2 (IQR: 1-4).

Most of the herds (137 of 168) owned by traders from which cattle were being sold were located in the Savannah Region, as well as other areas of Togo (5 of 168), Burkina Faso (22 of 168) and Ghana (4 of 168). Similarly, most of the herds (134 of 167) which received purchased cattle were in the Savannah Region, as well as other areas of Togo (5 of 167), Burkina Faso (22 of 167), Ghana (4 of 167), Benin (1 of 167) and Niger (1 of 167).

Cattle purchases and sales took place in 26 Savannah markets. Nearly three quarters of traders (152 of 213, 71.4%) bought cattle in at least one Savannah market and sold cattle in at least one other Savannah market. Around half of the cattle purchases and sales reported by the traders (53.5% and 51.3%, respectively) took place in Savannah markets, whilst 37.1% and 21.9% of purchases and sales took place in another country. Of the sales and purchases in Savannah markets, 82.4% were conducted in only 4 markets. Outside of Togo, most of the foreign cattle purchases (85.5%) occurred in Burkina Faso and 34.9% of foreign cattle sales took place in Nigeria.

More than half of the traders (116 of 213, 54.5%) operated in at least one other country outside of Togo. Among those traders operating in multiple countries, only one quarter (31 of 116, 26.7%) conducted both purchase and sale activities in at least two countries. Almost two thirds of the traders operating in Burkina Faso (49 of 81, 60.5%) conducted only purchases in Burkina Faso, without any sales. Half (11 of 22, 50.0%) of the traders operating in Ghana, three quarters (25 of 34, 73.5%) of the traders operating in Benin and all (27 of 27, 100%) of the traders operating in Nigeria only sold cattle in these countries, without purchasing. The number of traders operating at different purchase and sale locations as well as the numbers of cattle traded at these sites are summarised in Table A.

**Wet season cattle flow simulations**

During the wet season, 71.3% (range: 70.0-72.4%) of inflow into the Savannah market system was from Burkina Faso, with 7.9% (range: 7.6-8.3%) from Ghana and 3.1% (range: 2.8-3.5%) from Benin. Half of the cattle leaving the Savannah market system in the dry season (50.7%, range: 48.8-52.8%) were sent to Togolese markets outside of the Savannah Region. In terms of outflow from the Savannah market system, 8.9% (range: 8.3-9.5%) flowed into Ghana, 11.1% (range: 10.1-12.0%) into Benin and 14.1% (range: 13.0-15.2%) into Nigeria. The results of Location Scenario 2 did not demonstrate any major differences, as shown in the supplementary file Data S2.

In the wet season, 2,671 (range: 2,543-2,829) cattle flowed into herds in the Savannah Region, equating to 1.6% (range: 1.5-2.1%) of the estimated total cattle population size in the Savannah Region. Given that most herds likely breed their own replacement animals, many more animals flowed in the reverse direction from Savannah herds into the market system. There was a mean of 8,552 cattle (range: 7,899-9,363) leaving herds in the wet season, equating to 6.2% (range: 5.7-6.8%) of the estimated total cattle population size. Location Scenario 2 produced similar results with 2,667 cattle (range: 2,515-2,793) flowing into herds and 8,611 (range: 7,923-9,394) leaving herds. Flows into and out of the Savannah herds followed the same trends as the aforementioned market system flows.

**Market network in wet season**

The Savannah market system during the wet season consisted of 26 markets. They formed a well connected network incorporating all but one of the markets, such that the GWCC was 25 and nearly half of these markets (13) formed the GSCC. When using the alternative algorithm for reconstructing the order of market visits (see supplementary file Text S1), the GSCC was even higher, with a median of 18 markets (range: 13-22).

The majority of markets (16 of 26) received cattle from at least two other markets, with a maximum of 13 other markets. Approximately half of the markets (14 of 26) sent cattle to at least two other markets, with a maximum of 13. However, most cattle movements within the Savannah market network were mediated by a small number of markets: four markets accounted for 72.8% and 74.4% of the total weighted in- and out-degrees, respectively.

**Table A: Empirical data from interviews with 226 cattle traders – wet season**

|  | Purchase and sale locations | | | | | | | | | |
| --- | --- | --- | --- | --- | --- | --- | --- | --- | --- | --- |
|  | Savannah markets | Savannah herds | Savannah butchers | Other Togo markets | Other Togo herds | Benin | Burkina Faso | Ghana | Niger | Nigeria |
| No. of traders purchasing or selling* | 212 | 159 | 35 | 91 | 7 | 34 | 80 | 22 | 2 | 27 |
| No. of traders purchasing* | 189 | 151 | 0 | 16 | 6 | 9 | 79 | 11 | 0 | 0 |
| No. of traders selling* | 175 | 140 | 35 | 80 | 5 | 32 | 31 | 18 | 2 | 27 |
| No. of cattle purchased, ranging from min. to max.° | 53969-61868 (53.5%) | 6731-7520 (6.6%) | 0 | 2761-3094 (2.6%) | 329-358 (0.3%) | 2378-2835 (2.4%) | 32895-35746 (31.7%) | 3072-3339 (3.0%) | 0 | 0 |
| Median proportion (%) of total cattle purchased per trader^+^ (IQR) | 76.0 (53.0-99.0) | 2.0 (1.0-19.5.0) | 0 | 28.5 (19.0-34.5) | 2.5 (1.3-13.5) | 37.0 (23.0 -38.0) | 56.0 (29.5-94.5) | 39.0 (21.8-49.8) | 0 | 0 |
| No. of cattle sold, ranging from min. to max.° | 52176-58683 (51.3%) | 2470-2757 (2.4%) | 2606-3071 (2.6%) | 23177-26084 (21.8%) | 85-90 (0.1%) | 6042-6756 (5.9%) | 3492-4059 (3.5%) | 4936-5457 (4.8%) | 38-38 (<0.1%) | 7697-8851 (7.7%) |
| Median proportion (%) of total cattle sold per trader^+^ (IQR) | 85.0 (53.0-98.0) | 2.0 (1.0-4.0) | 15.0 (7.0-20.5) | 45.0 (30.8-61.3) | 2.0 (2.0-3.0) | 27.0 (20.8-33.0) | 4.5 (1.8-33.5) | 26.5 (20.3-38.5) | 7.0 (5.0-9.0) | 27.0 (19.0-99.0) |

* The numbers of traders purchasing from, and selling to, different locations over the wet season are shown. Markets and herds located outside of the study zone, the Savannah Region, are referred to as “Other Togo markets” and “Other Togo herds”, respectively. No butchers were located outside of the Savannah Region.

° The minimum and maximum numbers of cattle purchased and sold in these locations are presented for the dry season, expressed in brackets as percentages of the average number of cattle purchased or sold.

^+^ This is the median value of the proportion of each trader’s purchases or sales taking place in the given locations, expressed as a percentage. The interquartile range (IQR) is given in brackets. Traders that did not purchase in a given location were excluded.
