# Supplementary material for: Potential Risk of Regional Disease Spread in West Africa through Cross-Border Cattle Trade
Source: PLoS One. 2013 Oct 9;8(10):e75570. doi: 10.1371/journal.pone.0075570 (PMC3794041; doi:10.1371/journal.pone.0075570)
Supplement: Data S2 — Summarised simulation results for wet and dry seasons. The results of the 10,000 model simulations of livestock flows through the Savannah market system in the dry and wet seasons are provided in a table. This includes results for the independent and non-independent purchase and sale location scenarios, Location Scenario 1 and Location Scenario 2, respectively. (DOC) [file pone.0075570.s002.doc]

**Data S2: Summarised simulation results for wet and dry seasons**

The results of the 10,000 model simulations of livestock flows through the market system in the dry and wet seasons are summarised in the table below for the independent and non-independent purchase and sale location scenarios, Location Scenario 1 and Location Scenario 2, respectively.

*Cattle flow into the Savannah market system*: The data represent the mean proportion (%) of the total inflow into the Savannah market system from each of the given locations. The range is given in parentheses as minimum and maximum proportions.

*Cattle flow into Savannah herds*: The data represent the mean proportion (%) of the total inflow into Savannah herds from each of the given locations. The range is given in parentheses as minimum and maximum proportions.

*Cattle flow out of the Savannah market system*: The data represent the mean proportion (%) of the total outflow from the Savannah market system going to each of the given locations. The range is given in parentheses as minimum and maximum proportions.

*Cattle flow out of Savannah herds*: The data represent the mean proportion (%) of the total outflow from Savannah herds going to each of the given locations. The range is given in parentheses as minimum and maximum proportions

| **Cattle Flows** | **Origin or Destination** | | | | | | | | |
| --- | --- | --- | --- | --- | --- | --- | --- | --- | --- |
|  | Savannah herds | Savannah  butchers | Other Togo markets | Other Togo herds | Benin | Burkina Faso | Ghana | Niger | Nigeria |
|  | **Location Scenario 1 - Dry Season** | | | | | | | | |
| Into Savannah market system | 14.3  (11.9-17.7) | 0  (0-0) | 6.2  (5.8-6.9) | 0.3  (0.2-0.3) | 3.6  (3.1-4.7) | 68  (65.2-70.2) | 7.6  (7.2-8.0) | 0 | 0 |
| Out of Savannah market system | 5.7  (5.2-6.3) | 6.2  (5.6-6.9) | 49.3  (47.0-51.7) | 0.1  (0.0-0.1) | 12.3  (11.1-13.8) | 3.9  (3.5-4.4) | 7.8  (7.3-8.4) | 0.0  (0.0-0.1) | 14.7  (13.5-15.9) |
| Into Savannah herds | 18.1  (13.3-24.6) | 0  (0-0) | 6.4  (4.8-8.2) | 0.2  (0.0-0.5) | 5.7  (4.1-7.9) | 64.3  (58.8-69) | 5.3  (4.0-7.0) | 0 | 0 |
| Out of Savannah herds | 4.0  (2.5-6.4) | 4.9  (2.6-8.2) | 46.4  (39.4-54.7) | 0.0  (0.0-0.1) | 21.4  (16.0-25.7) | 1.5  (0.9-2.3) | 4.0  (2.6-5.8) | 0.1  (0.0-0.2) | 17.7  (11.4-25.5) |
|  | **Location Scenario 1 - Wet Season** | | | | | | | | |
| Into Savannah market system | 11.6  (10.6-13.0) | 0 | 5.7  (5.4-6.0) | 0.3  (0.3-0.4) | 3.1  (2.8-3.5) | 71.3  (70.0-72.4) | 7.9  (7.6-8.3) | 0 | 0 |
| Out of Savannah market system | 5.9  (5.4-6.4) | 6.5  (5.8-7.2) | 50.7  (48.8-52.8) | 0.1  (0.1-0.2) | 11.1  (10.1-12.0) | 2.7  (2.2-3.2) | 8.9  (8.3-9.5) | 0.0  (0.0-0.1) | 14.1  (13.0-15.2) |
| Into Savannah herds | 13.7  (11.0-16.8) | 0  (0-0) | 6.3  (4.7-7.9) | 0.2  (0.0-0.6) | 5.6  (4.2-7.2) | 68.5  (64.9-72.1) | 5.7  (4.1-7.6) | 0 | 0 |
| Out of Savannah herds | 4.3  (3.5-5.2) | 6.2 (5.3-7.3) | 48.5  (44.6-52.1) | 0.0  (0.0-0.1) | 19.1  (16.5-22.1) | 1.3  (0.9-1.8) | 5.8  (5.0-6.8) | 0.1  (0.0-0.2) | 14.6  (13.2-16.4) |
|  | **Location Scenario 2 - Dry Season** | | | | | | | | |
| Into Savannah market system | 17.6  (14.7-21.8) | 0 | 6.7  (6.0-7.7) | 0.2  (0.2-0.3) | 2.0  (1.5-3.0) | 65.6  (61.9-68.3) | 7.9  (7.4-8.4) | 0 | 0 |
| Out of Savannah market system | 7.5  (6.5-8.6) | 7.4  (5.7-8.8) | 47.9  (40.8-54.9) | 0 | 13.1  (9.9-16.8) | 1.7  (1.4-2.2) | 6.8  (4.4-9.6) | 0.0  (0.0-0.1) | 15.5  (10.8-20.2) |
| Into Savannah herds | 22.9  (16.1-29.7) | 0 | 5.0  (3.2-6.9) | 0.2  (0.0-0.5) | 1.6  (0.6-3.0) | 64.7  (56.7-70.7) | 5.7  (3.8-7.7) | 0 | 0 |
| Out of Savannah herds | 5.0  (3.1-7.9) | 5.6 (3.4-9.3) | 47.0  (39.7-59.0) | 0.0  (0.0-0.1) | 19.9  (13.0-25.6) | 0.6  (0.4-1.1) | 3.7  (2.0-6.3) | 0.0  (0.0-0.1) | 18.1  (10.8-26.3) |
|  | **Location Scenario 2 - Wet Season** | | | | | | | | |
| Into Savannah market system | 14.4  (13.1-16.2) | 0 | 6.0  (5.5-6.3) | 0.3  (0.3-0.4) | 1.4  (1.2-1.6) | 69.6  (68.0-71.2) | 8.3  (7.8-8.8) | 0 | 0 |
| Out of Savannah market system | 8.0  (6.9-9.0) | 7.8  (6.5-9.3) | 49.9  (43.4-55.5) | 0.0  (0.0-0.1) | 11.8  (9.0-13.9) | 0.5  (0.2-0.9) | 7.9  (5.1-10.8) | 0.0  (0.0-0.1) | 14.1  (9.4-19.1) |
| Into Savannah herds | 18.2  (14.5-23.0) | 0 | 4.6  (3.1-6.0) | 0.2  (0.0-0.6) | 1.0  (0.4-1.8) | 69.7  (64.3-74.6) | 6.2  (4.3-8.1) | 0 | 0 |
| Out of Savannah herds | 5.7  (4.5-7.1) | 7.2  (6.0-8.7) | 48.9  (44.0-54.0) | 0.0  (0.0-0.1) | 17.5  (14.3-21.2) | 0.2  (0.0-0.5) | 5.4  (3.5-7.5) | 0.0  (0.0-0.1) | 15.0  (11.6.19-0) |
