# Supplementary material for: Potential Risk of Regional Disease Spread in West Africa through Cross-Border Cattle Trade
Source: PLoS One. 2013 Oct 9;8(10):e75570. doi: 10.1371/journal.pone.0075570 (PMC3794041; doi:10.1371/journal.pone.0075570)
Supplement: Text S1 — Accounting for the order of market visits in the analysis of the Savannah market network. The process of stochastically ordering the market visits undertaken by traders in the Savannah market network is described for both dry and wet seasons. (DOC) [file pone.0075570.s003.doc]

**Text S1: Accounting for the order of market visits in the analysis of the Savannah market network**

**Methods**

The Savannah market network was constructed based on the results of the livestock flow simulations. The order in which the markets were visited by each trader was not known but could possibly influence the structure of the network. Potential orders of visits were constructed by running 1,000 stochastic simulations, whilst also ensuring that each scenario was plausible.

This process is best explained using a hypothetical example. Consider a trader who reports purchasing 100 cattle in Market A and 200 in Market B, and selling 10 in Market C and 290 in Market D. The first market visited by this trader must be one where he purchases cattle, i.e. either A or B. If Market B is randomly selected as the first market and 200 cattle are purchased, the second market could theoretically be either a purchase or sale location, i.e. Markets A, C or D. However, Market D is not plausible because the trader does not yet have the 290 cattle that he reports selling there. If Market A is randomly selected as the second visited market, the third market could either be Market C or D. If Market C is randomly selected, the final market visited is D. Therefore, although this trader may sell cattle in Market D which were purchased in the first market that he visited, Market B, these cattle were in fact moved through Markets A and C before reaching their final sale destination.

**Results**

The results of this ordered analysis are similar to those presented in the manuscript where the market visiting order was not taken into account, except for an increase in the giant strongly connected component (GSCC). As the GSCC is an estimate of the lower bound of the maximum epidemic size for a given disease, an increase in the GSCC reflects a greater potential for spread of the disease to other markets in the network.

*Dry season*

In the dry season, the market system consisted of 28 markets. They formed a well connected network incorporating all but one of the markets, such that the giant weakly connected component (GWCC) was 27 with most of these markets (median: 20, range: 14-23) forming the GSCC. The majority of markets (17 of 28) received cattle from at least 2 other markets, with a maximum of 10 other markets. Approximately half of the markets (16 of 28) sent cattle to at least two other markets, with a maximum of 11 other markets. However, most cattle movements within the Savannah market network were mediated by a small number of markets: 5 markets accounted for 75.5% and 79.8% of the total weighted in- and out-degrees, respectively.

*Wet season*

In the wet season, the market system consisted of 26 markets. They formed a well connected network incorporating all but one of the markets, such that the GWCC was 25 with most of these markets (median: 18, range: 13-21) forming the GSCC. The majority of markets (16 of 26) received cattle from at least 2 other markets, with a maximum of 10 other markets. The majority (16 of 26) also sent cattle to at least two other markets, with a maximum of 10 other markets. However, most cattle movements during the wet season within the Savannah market network were mediated by a small number of markets: 5 markets accounted for 77.2% and 79.9% of the total weighted in- and out-degrees, respectively.
